# Climax as Work: Heteronormativity, Gender Labor, and the Gender Gap in Orgasms

**DOI:** 10.1177/08912432211073062

**Published:** 2022-01-31

**Authors:** Nicole Andrejek, Tina Fetner, Melanie Heath

**Affiliations:** McMaster University, Canada

**Keywords:** orgasm, gender gap, gender labor, sexual behavior, heteronormativity

## Abstract

Gender scholars have addressed a variety of gender gaps between men and women, including a gender gap in orgasms. In this mixed-methods study of heterosexual Canadians, we examine how men and women engage in gender labor that limits women’s orgasms relative to men. With representative survey data, we test existing hypotheses that sexual behaviors and relationship contexts contribute to the gender gap in orgasms. We confirm previous research that sexual practices focusing on clitoral stimulation are associated with women’s orgasms. With in-depth interview data from a subsample of 40 survey participants, we extend this research to show that both men and women engage in gender labor to explain and justify the gender gap in orgasms. Relying on an essentialist view of gender, a narrow understanding of what counts as sex, and moralistic language that recalls the sexual double standard, our participants craft a narrative of women’s orgasms as work and men’s orgasms as natural. The work to produce this gendered narrative of sexuality mirrors the gender labor that takes place in the bedroom, where both women and men engage in sexual behaviors that emphasize men’s pleasure to a greater extent than women’s.

Gender scholars have addressed a variety of gender gaps between men and women from wages to household labor and workplace authority to political representation (e.g., England 2010; Stojmenovska and England 2021). There is also abundant evidence that a gender gap exists in orgasms between men and women during heterosexual sex (Armstrong, England, and Fogarty 2012; Brewer and Hendrie 2011; Wade, Kremer, and Brown 2005). Although sexuality can feel personal and intimate, the persistent orgasm gap suggests that a social dynamic is at play. We consider how gender and heteronormativity affect sexual relationships in ways that maintain inequality in the realm of heterosexual sex.

While we may imagine that traditional gender scripts are receding, there is evidence that gender inequality persists, especially in heteronormative contexts. For example, although men are increasing their contributions to the home, women are often still responsible for the majority of domestic work such as house cleaning and child rearing (England 2010). In part, this division of labor may be attributable to cultural narratives—and even essentialist ideas—about how men and women are expected to behave (Perry-Jenkins and Gerstel 2020). Similar dynamics are at play in intimate relationships between women and men. In her study of dating practices, Lamont (2014) finds that women want egalitarian relationships but often rely on common traditional gender stereotypes and essentialist beliefs about men and women. Women reframe their own beliefs to reconcile inherent contradictions between what they want from a relationship and their reality, reframing men’s inegalitarian behaviors as romantic. These gender scripts shape and limit women’s expectations and practices.

Research on the gender gap in orgasms in heterosexual, partnered sex provides important insights into the determinants of women’s orgasms (Armstrong, England, and Fogarty 2012; Laumann et al. 1994). In particular, sexual practices focused on clitoral stimulation, such as oral sex, are particularly important to reducing the gender gap in orgasms (Shirazi et al. 2017). However, an important question persists: If it is widely known and accepted that clitoral stimulation enhances women’s sexual pleasure, why are couples not engaging in the types of sexual activities that might reduce the gender gap in orgasms?

We argue that, to comprehend why the gender gap in orgasms persists in heterosexual sexual encounters, research is needed on the meanings that individuals place on sex and orgasms. Building on theories of the “gender labor” that heterosexual couples do to make gender inequalities in their relationships appear natural (Ward 2020, 89), we examine the narratives adults produce to communicate their understandings of their bodies, sexuality, and different sexual practices, to illuminate the work that men and women do, and to justify and make sense of gender differences in orgasms. Through this approach, we move past individualistic explanations of the gender gap in orgasm to examine how adults’ sex lives reflect and maintain broader gender inequalities.

This article extends the research on the gender gap in orgasms in two ways. First, we expand beyond studies of young adults in the American college context with an original survey of sexual behavior of Canadian adults, allowing us to analyze a representative sample of adult heterosexual relationships, whether they are casual, committed, or something in between.^1^ Next, we investigate the heteronormative and gendered understanding of sex, orgasm, and pleasure through both survey analysis and in-depth qualitative interviews. By examining our participants’ narratives concerning sex and gender differences in orgasms as a form of gender labor, we demonstrate how the gender gap in orgasms persists in heterosexual sexual encounters.

## Sociological Approaches to Orgasms

A gender gap in orgasms is a consistent, long-standing finding across many studies that examine heterosexual sexual encounters (Armstrong, England, and Fogarty 2012; Darling, Davidson, and Cox 1991; Klassen and Wilsnack 1986). In sex between men and women, men report having an orgasm more than 90 percent of the time, but women’s orgasm rates are much lower (Brewer and Hendrie 2011; Richters et al. 2006; Wallen and Lloyd 2011). Wade, Kremer, and Brown (2005) found that 39 percent of women compared with 91 percent of men report usually or always experiencing an orgasm during partnered sex. More recently, Frederick and colleagues (2018) found a rate for women of 65 percent, compared with 95 percent of men—still a significant gap.

Previous anatomical explanations for why women are less likely to orgasm have been repeatedly debunked. Those explanations relied on the embodied aspects of sexuality that can reify essentialist, biological explanations that men are anatomically easier to please than women (Armstrong, England, and Fogarty 2012). Research has disputed this explanation, employing a variety of evidence such as women’s higher orgasm rates when engaged in same-sex sexual encounters and ability to consistently reach orgasm through masturbation (Carvalheira and Leal 2013; Frederick et al. 2018).

Women also report more sexual satisfaction during partnered sex in which they had an orgasm, refuting notions that orgasms are inconsequential to women’s sexual enjoyment (Waterman and Chiauzzi 1982). Corollary to the idea that women’s bodies are difficult to please is the claim that women’s skill-building through masturbation will lead to more orgasms in partnered sex; however, this claim has not been supported by empirical research (e.g., Andrejek and Fetner 2019).

Rather, two primary mechanisms have been identified through which the gender gap in orgasms is produced: relationship context and sexual practices (Armstrong, England, and Fogarty 2012). The first explanation focuses on the relationship context of sexual partners during heterosexual sex (Sanchez et al. 2011). Men and women report higher levels of sexual satisfaction and higher rates of orgasms in the context of committed relationships compared with casual sexual encounters (Holmberg and Blair 2009; Mark, Garcia, and Fisher 2015; Richters et al. 2006; Waite and Joyner 2001). The second focuses on sexual behavior and clitoral stimulation. Research consistently shows that engaging in a variety of sexual practices that stimulate the clitoris significantly increases women’s likelihood of having an orgasm (Darling, Davidson, and Cox 1991; Herbenick et al. 2018; Laumann et al. 1994; Prause et al. 2016; Richters et al. 2006). Given that the clitoris is the pleasure center for female sexuality, it makes a great deal of sense that sexual behaviors that stimulate it will produce more orgasms. Indeed, the findings associating relationship context with increased orgasms for women may simply be identifying the conditions under which sexual interactions between men and women are more likely to include clitoral stimulation. For example, committed partnerships may lead to increased time for men to learn about their partners’ desires, become more skilled in pleasing them, and feel more accountable for their partners’ pleasure (Laumann et al. 1994; Waite and Joyner 2001). Individuals in committed relationships also tend to engage in a greater variety of sexual activities than those engaged in casual sexual encounters (Babin 2013; Herbenick et al. 2010).

## Heteromasculinity, Gender Labor, and the Orgasm Gap

Both explanations point to the role of larger social forces in organizing and regulating gender relations in heterosexual contexts. Heterosexuality as a patriarchal institution promotes commonsense understandings of what constitutes sexual pleasure for cisgender women and men, prioritizing men’s sexual pleasure and orgasms over women’s (Ward 2020). Hegemonic masculinity, in particular, proffers a heteromasculine ideal of men’s sexual prowess based on virility and skilled performance. Montemurro (2021) found that being able to satisfy a woman’s sexual appetites and desires—especially by “giving” orgasms—was high on the list. However, the same heteromasculinity contributes to a “phallocentric imperative” that undermines women’s claims to clitoral stimulation (Wade, Kremer, and Brown 2005; Willis et al. 2018). Cultural narratives of masculinity are closely associated with sexual practices, such as in the relationship between heterosexual men’s willingness to claim a feminist identity and their greater likelihood of performing oral sex on women partners (Stick and Fetner 2021). Heterosexual men, especially those who occupy dominant racial categories, may simply feel more entitled to orgasms than their women partners do (Jones 2018; Manne 2020). In addition, the sexual double standard, which imposes shame upon women for the same behavior as men, influences the perceptions concerning expectations about heterosexual sex, as well as perceptions of women’s right to sexual pleasure (Armstrong, England, and Fogarty 2012; Salisbury and Fisher 2014; Tolman 2012).

Representations of penile–vaginal intercourse in the broader cultural consciousness emphasize penile stimulation and de-emphasize clitoral stimulation. Cultural representations of sex—in sexually explicit materials such as pornography and romance novels, as well as sexually suggestive materials such as mainstream film, television, and magazine articles—often portray sexual encounters in which women easily reach orgasm from penile–vaginal intercourse alone (Klaassen and Peter 2015; Porter, Douglas, and Collumbien 2017; Séguin, Rodrigue, and Lavigne 2018). Penile–vaginal intercourse is portrayed—and understood—as the main event in heterosexual encounters. In this way, other sexual activities that place greater emphasis on clitoral stimulation, such as oral sex, are characterized as something other than regular sex, categorized as foreplay that happens before sex or as a special treat that is considered optional. This sexual script places an expectation upon women to orgasm in a timeframe oriented toward men’s pleasure, which can lead women to fake orgasms to protect the feelings of their partner (Muehlenhard and Shippee 2010).

The work that men and women do to create a sex life that conforms to dominant narratives of “normal” sexuality can be seen in terms of what Jane Ward (2010, 2020) calls “gender labor”: the effort necessary for both heterosexual desirability and the gender binary to appear natural. This labor harnesses heteronormative beliefs about what counts as real sex, what kinds of sex are good and right, and what is dirty and suspect (Schilt and Westbrook 2009; Ward 2020). In the following, we argue that to understand the continuing gender gap in orgasms, we must theorize the work that women and men do to maintain a gender hierarchy and to uphold heteronormativity while rendering both of these as natural and taken for granted. Thus, the gender labor that heterosexual couples do in other areas of their relationship continues into their sexual lives. We demonstrate how the orgasm gap is fundamentally based on a form of gender labor that makes men’s orgasms appear normal and ordinary, in contrast to women’s orgasms that are seen as anomalous. Below, we elaborate our mixed-methods approach to unpacking this problem.

## Data and Methods

We use an original survey of sexual behavior, with follow-up interviews, to explore these questions fully. Our analysis of survey data establishes the presence of an orgasm gap among heterosexual Canadian adults, extending these findings beyond studies of university students and other young adults. Restricting our sample to women having sex with men, we employ logistic regression analysis to test three predictors of whether women had an orgasm in their most recent sexual encounter. Our analysis of in-depth interviews of a subsample of women and men survey participants explores the strategies that participants employ to make sense of their own sexual behavior and that of their partners.

### Analysis of Survey Data

We analyze quantitative data from an original survey of a representative sample of adult (18+ years old) Canadians, compiled by a stratified sampling strategy that used quota-based sampling strategy to match census demographics of the Canadian population. Survey participants were recruited by Environics Canada from their proprietary panel of 400,000 research participants, a large pool of volunteers who receive proprietary rewards for completing surveys of all kinds. From this pool, Environics drew a subsample that proportionally matched the Canadian population, according to the most recent census, on the following characteristics: gender, age groups, visible minority status (a Canadian indicator that asks individuals to self-identify as someone who feels racially marked by others—most often including Black, Latinx, Asian, and mixed-race people), primary language (English or French), highest level of education, and region of residence.

A sample of 2,303 participants recorded their survey responses through an online survey tool, which allowed for anonymity and maximum privacy. No identifying information was collected. The survey questionnaire instrument was a modified version of the questionnaire used by the U.S.-based National Survey of Sexual Health and Behavior (Herbenick et al. 2010). This research involving human participants was approved by McMaster University Research Ethics Board, project #2017-113. All participants gave fully informed, written consent to participate.

Quota-based, nonprobability samples of online survey panels are widely used in social science today, because they maximize population representativeness in the current social context, where survey response rates for random population sampling are low (Gosling et al. 2004). For topics such as sexual behavior, which have largely been ignored by large-scale probability surveys with random sampling, nonprobability sampling by quota is an important alternative approach (Elliott and Valliant 2017). Some concerns for bias may include under-coverage of the population due to a reliance on Internet users, who differ from the population at large in terms of age, income, and region (Thompson and Pickett 2020). Our sample mitigates some of these potential biases by including age and region as quota criteria. Another source of bias may be self-selection of those willing to complete an anonymous survey of sexual behavior, which may produce an overrepresentation of those who are more comfortable with sexual topics than the general population.

### Dependent Variable

Our dependent variable is a self-report of orgasm in the participant’s most recent sexual encounter. The question reads, “During this most recent sex act, did you have an orgasm?” The participants were given the option to respond yes, no, or “I don’t know/don’t remember.” For the analysis, we combined the “no” and “I don’t know/don’t remember” responses to distinguish affirmative memories of orgasm from other sexual experiences.

### Focal Independent Variables

Our analysis centers on three focal variables that the literature on the orgasm gap has identified as the primary explanations for the gender gap in orgasm:

#### Masturbation

Related to the myth of the female body being more challenging to bring to orgasm than male bodies, previous research has considered whether women’s practice of masturbation is related to their likelihood of reaching orgasm in partnered sex. The thinking is that women who masturbate more frequently have more fluency in what brings their body to orgasm (Wade, Kremer, and Brown 2005). To date, there has not been much support for this relationship (Andrejek and Fetner 2019); however, we examine this relationship on our nationally representative sample through the question “how recently have you done the following? I masturbated alone,” in which we compare those who masturbated within the past 30 days to all others.

#### Relationship to Sexual Partner

As stated above, women report having more orgasms with committed partners than with those to whom they have a more distant or unclear relationship (Mark, Garcia, and Fisher 2015). We measure relationship to sexual partner with participants’ responses to the question “what was your relationship to the person you had sex with in your most recent sexual encounter?” We compare those whose sexual partner is a spouse or a common-law partner (with whom one has shared a residence for at least one year) with all other relationship types.

#### Receiving Oral Sex

Our measure of sexual behaviors targeting clitoral stimulation is a yes/no indicator that the participant received oral sex. This is part of a suite of questions on sexual behaviors related to their most recent sexual encounter, worded “During this sexual experience, which of the following activities occurred?” with a “check all that apply” list of sexual behaviors, such as *we kissed, we cuddled*, and *we had penile–vaginal intercourse*. Among this list, two items are related to clitoral stimulation: “We used our hands to stimulate each other’s genitals (partnered masturbation or ‘hand jobs’)” and “My partner gave me oral sex.” Because the first measure does not distinguish between penile and clitoral stimulation, we elect to use the measure “My partner gave me oral sex” to capture clitoral stimulation. We acknowledge that a wide variety of sexual behaviors produce clitoral stimulation, including manual stimulation and vibrators. While we are able to include only oral sex in the present analysis, we anticipate that all clitoral stimulation techniques will increase the likelihood of orgasm for women.

### Control Variables

#### Age Group

As sexuality and sexual experiences change over the life course, there has been a consistent association between age and sexual behavior that may affect women’s orgasms. Participants were divided into age groups that roughly approximate life-course stages: (1) 18–24 years (reference), (2) 25–34, (3) 35–44, (4) 45–54, (5) 55–64, and (6) 65 and above.

#### Education Level

Education is a consistent predictor of sexual behavior. Level of education of participant: (1) less than a high school diploma (reference), (2) high school diploma, (3) 2-year college or trade school, or (4) university degree or higher. In Canada, the 2-year college and trade system is akin to community colleges in the United States, oriented primarily around 2-year diplomas, job training programs, and preparation for skilled trades.

#### Religious Attendance

Because religious institutions often seek to regulate sexual behavior of their constituents, religiosity has often been found to be associated with sexual practices. Religiosity is measured by self-reported attendance at religious services: (1) never (reference), (2) less than once a month, (3) once or twice a month, or (4) weekly or more.

### Analytic Strategy

Our quantitative analytic strategy is twofold. We first establish a gender gap in orgasm in heterosexual encounters using a Fisher’s exact test to determine whether the difference between women and men in the likelihood of orgasm is statistically significant. This analysis relies on a subsample of *n* = 1,798. Given that sexual identity and sexual behavior are sometimes dissonant (e.g., Silva 2017, 2021) rather than rely on sexual identity, we include men whose most recent sexual partner was a woman, as well as women whose most recent sexual partner was a man.

Our second analytic strategy uses logistic regression to test three hypotheses predicting women’s orgasm:

**Hypothesis 1:** a *skills* hypothesis: Women are more likely to have an orgasm if they are more practiced at masturbation alone;
**Hypothesis 2:** a *relational* hypothesis: Women are more likely to have an orgasm if their sexual partner is their spouse or common-law partner;
**Hypothesis 3:** a *behavioral* hypothesis: Women are more likely to have an orgasm if their sexual partner performs oral sex.

To do this, we further limit our subsample to women only. We include those women whose partner was a man and who completed the survey questions related to their most recent sexual encounter. This produced a subsample of *n* = 950. We employ logistic regression models to estimate a binary dependent variable: the presence or absence of women’s orgasm in their most recent sexual encounter. Model 1 considers the relationship between recent masturbation with women’s orgasm. Model 2 examines whether a woman’s sexual partner in her most recent sexual encounter was a spouse/common-law partner. Model 3 examines the relationship between receiving oral sex and orgasm. Finally, Model 4 includes all focal variables to offer a robustness check. Analyses produce log-odds coefficient estimates for probabilities that a woman participant reported an orgasm in her most recent sexual encounter with a man.

### Analysis of Follow-Up Interviews

We analyze in-depth interviews of a subsample of survey participants who identify as men (*n* = 20) and women (*n* = 20). At the end of the survey, respondents were given the option to be contacted to participate in a follow-up interview. Interviews were conducted using a video-conferencing platform or over the phone, depending on participants’ preference. Each interview lasted approximately one hour, during which participants were asked about their current relationship and relationship history, their sexual history, and their attitudes and perspectives on sexualities-related topics. We used a semi-structured interview guide in a conversational manner to encourage participants’ comfort discussing these intimate and sensitive topics. The participants in our sample are predominantly white, and, while many identify with a current religion, the majority are not currently active (for detailed participant characteristics, see the Online Appendix A1). Interview transcripts were coded using interpretive methods of analysis, facilitated through the qualitative software Atlas.ti. We developed a coding protocol to use techniques of thematic analysis to reveal common themes based on previous research in the field and explanations of changing understandings of sexual practices (Braun and Clarke 2012).

## Findings

### Survey Analyses

Table 1 summarizes the gender gap in orgasms among those participants whose most recent sexual encounter was heterosexual. Consistent with previous studies, we find that women are less likely than men to report that they experienced an orgasm in their most recent sexual encounter. Among men who had sex with a woman in their most recent sexual encounter, 86 percent report having had an orgasm. Among women who had sex with a man, 62 percent report having had an orgasm. A Fisher’s exact test shows that this gap of 24 percentage points is statistically significant (*p* < .001).

**Table 1:** The Gender Gap in Orgasms Among Participants With Other-Gender Partner

	*N*	Percent reporting orgasm in most recent sexual encounter
Men	848	86***
Women	950	62***

Fisher’s exact test: *p < .05. **p < .01. ***p < .001.

Next, we consider three explanations for women’s likelihood to experience orgasm. We limit the following analyses to women whose most recent sexual encounter was with a man. Table 2 shows the frequencies of women participants who report having had an orgasm in their most recent encounter by each of the three dependent variables: frequent masturbation, a spouse or common-law relationship to their sexual partner, and having received oral sex.

**Table 2:** Frequency of Women Reporting an Orgasm by Masturbation, Relationship to Sexual Partner, and Receiving Oral Sex

	Percent with orgasm
Masturbated this month	
Yes	63.1
No	62.1
Relationship to sexual partner	
Spouse/common-law partner	63.6
Other relation	59.4
Received oral sex	
Yes	72.9***
No	57.0***

Fisher’s exact test: *p < .05. **p < .01. ***p < .001.

Table 3 summarizes the results of our estimates of coefficients using logistic regression analysis, our tests of three hypotheses. Model 1 tests the *skills* hypothesis, which posits that practice through masturbation increases the likelihood that women will reach orgasm in partnered sex. Model 2 tests our *relational* hypothesis, which proposes that women are more likely to experience an orgasm in a committed relationship, such as with a spouse or common-law partner. Model 3 tests the *behavioral* hypothesis, which posits that those sexual behaviors which stimulate the clitoris are more likely to produce an orgasm for women. Finally, Model 4 includes all focal independent variables in a full model that considers each of these explanations.

**Table 3:** Logistic Regression Estimates for Women’s Orgasm in Most Recent Sexual Encounter

	Model 1	Model 2	Model 3	Model 4
Masturbated in last 30 days				
No (reference)				
Yes	–0.020			–0.045
Relationship to recent partner				
Other relation (reference)				
Spouse/common-law partner		0.320		0.374
Received oral sex				
No (reference)				
Yes			0.923***	1.020***
Age (years)				
18–24 (reference)				
25–34	0.276	0.143	0.411	0.139
35–44	0.364	0.243	0.641	0.301
45–54	0.490	0.319	0.558	0.270
55–64	0.301	0.277	0.687	0.284
65+	0.531	0.427	0.858	0.558
Education				
Less than HS (reference)				
HS diploma	–0.033	–0.216	–0.158	0.033
2- Year college or trade school	0.239	0.116	0.134	0.284
University degree or higher	0.439	0.290	0.304	0.516
Religious attendance				
Never (reference)				
Less than 1×/month	0.305	0.379	0.347	0.371
1–2×/month	0.470	0.627	0.560	0.511
Weekly or more	0.478	0.561*	0.659*	0.661*
Constant	–0.267	–0.338	–0.764	–0.899
Chi-square	11.648	16.752	37.222	39.636
Akaike info. criterion	775.482	817.003	800.367	747.665

Note: HS = high school; × = times.

* *p* < .05. **p < .01. ***p < .001.

Our results support neither the *skills* hypothesis (Model 1) nor the *relational* hypothesis (Model 2). We find no significant association between either having masturbated within the past month (*b* = –0.02, *p* > 0.05) or having had sex with a spouse or common-law partner (*b* = 0.320, *p* > 0.05). Our findings do offer strong support for the *behavioral* hypothesis (Model 3) that women who receive oral sex are more likely to experience orgasm (*b* = 0.923, *p* < 0.001). The strong positive association between having received oral sex and orgasm in women is statistically significant. This finding is further confirmed by Model 4, which includes all focal independent variables and offers the best fit of the data. The takeaway message from our quantitative analysis, consistent with a broad range of literature on the gender gap in orgasms, is that the gender gap in orgasm remains primarily associated with a lesser emphasis on clitoral stimulation.

We note that, for the most part, our control variables are not significant predictors of women’s orgasms. Age, which is so often associated with sexual behavior, is not associated with women’s orgasm, nor were our supplementary interaction terms between age and frequency of masturbation, relationship to partner, or having received oral sex (not shown). Neither is education significantly associated with women’s orgasm in any of our models. Religious attendance in the “weekly or more often” cate- gory is significant in three of our four models, which indicates that this group differs significantly in their likelihood of experiencing orgasm compared with those who never attend religious services.

### The Gender Gap in Orgasms Persists, But Why?

Our findings, which support the behavioral hypothesis that women’s orgasms are associated with clitoral stimulation, confirm previous research and extend it to a nationally representative sample. However, this finding raises as many questions as it answers. Foremost, why do sexual encounters between men and women sometimes fail to include clitoral stimulation? How do women and men make sense of their sexual interactions in a way that justifies a greater focus on men’s pleasure? To understand why the gender gap in orgasm persists in heterosexual sexual encounters, we examine the orgasm gap as a form of a gender labor, identifying three forms that emerged through our discussions with adults describing sexual encounters in heterosexual contexts. Below, we consider three elements of gender labor that our participants engaged in to understand their own and their partners’ understanding of sex and sexual pleasure.

### Crafting Essentialist Rationales for the Gender Gap in Orgasms

Gender labor refers to the work individuals do to reinforce one’s masculinity or femininity. In our interviews, we saw patterns of gender labor across many of our participants’ explanations for men’s and women’s orgasms (or lack thereof). Our participants’ depictions reveal how both men and women are doing work to reproduce traditional gender norms in which women are expected to prioritize emotional, romantic intimacy and men to prioritize physical sexual desire and pleasure. This work is done through narratives that offer essentialist explanations of whose pleasure is natural in heterosexual sex. Therefore, our participants’ justifications of the gender gap in orgasms are deeply connected to other essentialist justifications for gender inequalities in heterosexual relationships.

Our participants explain why men orgasm more than women by drawing on traditional, essentialist gender beliefs about men’s sexuality. Both men and women describe men’s orgasms as deeply tied to their masculinity and as the purpose of sex for men. For instance, when asked about how often he has an orgasm, Carter said “Cause I’m a guy, it’s mandatory . . . That’s almost a redundant question.” Similarly, Kathy expressed that “I have not known a man that has not had [an orgasm] with sex. I don’t think [a man] would have sex if he thought he couldn’t [orgasm].” Both men and women in our interviews characterized men’s orgasms as natural and obvious. Jane also said, “I think men . . . *need to orgasm* . . . But I don’t feel like I need [to] orgasm.” Ashley agreed:

For most men, [having an orgasm] is the main goal. For women, they just want it to be a good time and feel appreciated and not be painful, and women, well most women, are a lot less hung-up on the actual orgasm in my experience.

Ashley’s words underscore the low expectations that many women articulate about sex, such as that it should not be painful.

Both men and women expressed great concern for men’s orgasms, seeing them as very important to men’s sexual experience. Indeed, they reacted with great concern to the idea that a man may not orgasm, describing sex without a man’s orgasm as not just highly abnormal, but likely due to them having a physical or mental ailment. Chris said “If you’re not having one, there’s something wrong physically.” Men participants who confessed that they sometimes did not reach orgasm attributed this to a physical or mental illness. For example, Bob explained that the only thing affecting his ability to orgasm was “extreme stress.” Alex described an emotionally distressing cycle of not being able to orgasm due to stress, becoming more distressed over failing to orgasm, his wife blaming herself, and perpetuating these negative feelings and expectations in future sexual encounters. The collective work of crafting a narrative in which men’s orgasms are natural and obvious, and are connected to men’s sense of self and masculinity, means that being unable to orgasm can be emotionally painful and embarrassing. Because women also believe that it is easy for men to orgasm, this expectation can lead women to feel blameworthy if their partner does not orgasm.

In contrast, our participants portrayed women’s lack of orgasms as normal and expected. Their narratives characterize what women “naturally” want from sex as *emotional intimacy* instead of physical pleasure. Women’s orgasms are deemphasized in favor of a story that is more relational and emotional, drawing on traditional gender stereotypes about women’s natural affinity for caring and a desire for romance. For instance, Kathy said that she thought men have an inherent need to orgasm during sex but that “for a woman, it’s different. Great if I do, but if that emotional connection’s there [that’s what I need]. I think [an orgasm is] just something a man needs a lot more than a woman.” Similarly, Ashley said “I do not think I ever had [an orgasm] in my first relationship and we were together for five years. I don’t need to have an orgasm to enjoy having sex.” For her, “sex is not about it feeling good, it’s about being with somebody.” Ashley highlighted a common theme in which women framed their emotional needs as more important than their physical pleasure. Anthony expressed a similar sentiment:

Men prioritize orgasms more than women because it provides that sense of joy and pleasure. Whereas with women, I feel like that emotional side of getting to be intimate with the person is more important than giving them an orgasm.

In this narrative, both participants are responsible for men’s orgasm, which generally occur naturally, but neither partner is in charge of ensuring women’s sexual pleasure. Our participants’ narratives reveal the work they do to naturalize men’s orgasms as a need, while devaluing women’s orgasms as secondary to an emotional connection. Both the men and women in our study draw on stereotypical gendered conceptions of women’s sexuality when describing the purpose of sex for women, coming to the rationale that men and women are simply different from one another.

But is it truly the case that women have less desire to orgasm during partnered sex? Or that men and women have natural differences when it comes to their motivations to have sex? Our findings point to the fact that men and women’s limited expectations for women’s orgasms have less to do with women’s inherent inability or lack of desire to orgasm but to the norms of heterosexuality and gender that limit and confine expectations along gender lines. Through their descriptions of the purpose of sex for men and women, our participants show the collective work of sustaining these perceived essential gender differences. Ascribing men to the physical and women to the emotional as natural gender differences in sexual encounters is remarkably similar to the rationales that have been used to justify other gender gaps in heterosexual relationships, such as in childrearing and household labor (England 2010).

### Constructing the Definition of Sex in Narrow, Phallocentric Terms

Another element of gender labor that our participants engage in to justify the gender gap in orgasm is to define what counts as sex in narrow, phallocentric terms. The idea that “regular sex” refers to vaginal–penile intercourse was dominant in our interviews. For instance, when asked about the sexual practices that Sarah enjoys while having sex with her partner, she replied “I just like just the two of us together. *I just like regular intercourse*.” Jenn also stated that “the missionary or doggie style’s fine … I guess I just like *regular sex* . . . There’s usually only oral sex for our birthdays.” By defining “regular sex” as penile–vaginal intercourse, our participants make the case that regular sex is centered on stimulation of the penis, rather than the clitoris. This heteronormative and phallocentric understanding of what it means to have sex emphasizes penile stimulation, while relegating any sexual behaviors that focus on the clitoris as special, outside the boundaries of “regular sex.”

Our participants craft narratives that define regular sex as only penile–vaginal intercourse and sexual behaviors that prioritize clitoral stimulation, such as oral sex, vibrators, or manual stimulation, as “alternative” sexual practices. These alternative sexual practices to regular sex are depicted as more time-consuming labor and extra work for couples. Seen through the lens of phallocentric sex, women’s orgasm is characterized as unnecessary and challenging. In contrast, women’s orgasms are characterized as outside the bounds of regular sex, involving “more work” that is “too time consuming.” Charles said, “It’s definitely easier for the male, that’s for sure. I think [for the] female, it takes more work and *certain things have to be done*, where a male is good for anything.”

In defining what counts as regular sex so narrowly, our participants engage in gender labor that casts men’s orgasms as easy and quick, and women’s orgasms as difficult and time-consuming. Although some sexual practices may be more likely to bring women to orgasm, the fact that these were viewed as labor-intensive meant that they were often sidelined. For instance, Kathy said she enjoys

if the man is behind me and he is able to pleasure me with his hands [but it takes] *a lot more to work*. It takes *a lot more* for me to get to that point where I’m going have an orgasm.

Kathy’s comments highlight how women often prioritize men’s comfort over their own sexual pleasure.

This narrow, phallocentric definition of sex also produces a timeline for heterosexual encounters. When sex begins with penetration and ends with men reaching orgasm, there is a timeframe to sex that leaves little room to accommodate women’s orgasms. Many of our participants claimed a preference for both sexual partners to reach orgasm, but expressed that there wasn’t sufficient time for women partners to climax. For example, Rachel explained that the timeline of sex with her partner does not allow her to orgasm, whereas she is able to do so when masturbating:

More than 90 percent of the time, I honestly never orgasm . . . because it just happened too soon for him and then . . . it’s . . . not the best physical experience. I think he just doesn’t touch me long enough for me to be able to get there.

Jenn informed us that “mostly men are so quick, that it’s over before you even had a chance to get stimulated.” The men in our interviews also agreed. When asked about how often his partner orgasms, Charles replied:

My partner? Not always . . . I have a timeframe and unfortunately her timeframe is a lot longer than my timeframe. So I’ll always have to go first and then we might have to do it again, but her timeframe starts from zero again, so you have to pretty much reverse the roles and say, “Well, we’re going to focus on you having an orgasm, not me . . . .” Because you always care about that, right?

Similarly, Henry explained that

I try to make [my partner’s orgasm] happen, but it doesn’t seem to all the time . . . . My concern is how do I prevent [my orgasm] from happening? I would wish it wouldn’t happen so easily. I’m not saying I’m a premature ejaculation guy, but it’s like, Jesus Christ, sometimes you wish it would last a little longer.

The gender labor of upholding a definition of regular sex centers and normalizes men’s sexual pleasure, while marginalizing and problematizing women’s pleasure. This fits neatly with the essentialist view that we discussed in the previous section: that orgasms are more important to men than women. Next, we turn to a third element of gender labor: crafting the types of sexual practices focused on clitoral stimulation as kinky, dirty, and wrong.

### Producing Moralistic Judgments of Women’s Sexual Pleasure

The final element of gender labor supporting the gender gap in orgasms came from a subset of our women interviewees, who expressed discomfort with the idea of reaching orgasm with a sexual partner. These women described a belief that alternative practices to penile–vaginal intercourse are unnatural, bad, or dirty, invoking a sexual double standard that places a moralistic judgment on women’s sexual pleasure. Some women in our study described feeling badly after participating in what they characterize as alternative sexual practices, even if these were pleasurable and more likely to bring them to orgasm.

Sexual behaviors, such as watching pornography, giving or receiving oral sex, and using vibrators or sex toys, were described as not only separate from regular sex but also morally contentious. Ashley explained,

I’ve had a couple of boyfriends that were kind of kinky and wanted to do all these weird things and wanted to buy me sex toys [but] that’s not something I’m into. [I] don’t own dildos and vibrators because I want to be [emotionally intimate] with somebody.

This view that using a vibrator makes emotionally intimacy impossible is a form of self-regulation that precludes sexual practices that would make orgasm more possible, despite that using a vibrator might be beneficial for clitoral stimulation and reaching orgasm during partnered sex. Kathy told us that she needs clitoral stimulation to orgasm, but that

I don’t do oral sex . . . It can be very pleasurable, but it feels wrong to me . . . It just doesn’t feel natural . . . . I feel like it’s dirty sex, almost like watching porn. I’m not into porn and I’m not into oral [sex], it just makes me feel dirty.

The idea of what counts as regular sex is related to what our participants view as morally wrong or unnatural. By distinguishing oral sex from regular sex, participants are able to mark the former as “other” and wrong, while feeling good about the latter. These descriptions about some sexual practices as “dirty” or “not natural” also reflect the impacts of the sexual double standard on women’s perspectives about their sexuality and bodies. For instance, it is likely that Kathy’s feelings about oral sex being “dirty” derive from broader cultural and moralistic myths that have contributed to hegemonic notions that women’s bodies and sexuality are gross, shameful, or sinful.

By crafting a moralistic narrative of the types of sexual behavior that are more likely to bring women to orgasm, our participants highlight how traditional gender beliefs shape women’s perspectives about their bodies, based on a sexual double standard that views some sexual practices as slutty, shameful, and kinky. In general, only a few women discussed oral sex or clitoral stimulation as important to them, and many described closeness and intimacy as more important than physical pleasure. Although clitoral stimulation may bring them pleasure, some see a moral cost to participating in sexual practices such as oral sex. These findings reveal the resilience of the sexual double standard in making many women uncomfortable about so-called alternative sexual behaviors.

The traditional, essentialist gender beliefs—and resulting lack of expectations—about sex that we have addressed thus far also contribute to a culture of shame regarding women’s sexuality that is deeply tied to the institution of gender and heterosexuality. These moral meanings uphold the same system, though they are a different method of gender labor. Women are doing the work of explaining their lack of orgasms as a moral, individual choice. Women’s shame about sex (and their bodies) and the silencing of what they want in their sexual experiences is deeply gendered in that it maintains men’s privilege and power and is also deeply and inextricably tied to the institution of heterosexuality. Our participants are doing this gender labor to make sense of why women are not having orgasms as often as men.

## Conclusion: The Gender Labor of Heteronormative Sex

Gender labor makes men’s orgasms appear natural—an essential and expected aspect of sex—in contrast to women’s orgasms—thought of as work and difficult to achieve. Although the gender gap in orgasms might seem like an intimate problem exclusive to one’s bedroom, our quantitative and qualitative findings demonstrate that the gender gap in orgasms is not an individualistic issue of sexual function and dysfunction. Instead, it is enabled by the institutions of heterosexuality and gender, which encourage both men and women to do labor that makes gender differences appear natural and reproduces gender inequalities in heterosexual relationships.

In this article, we examined a representative sample of heterosexual Canadians to find that, as in previous research, there is a gender gap in orgasms. Sexual practices focused on clitoral stimulation are key to reducing this gap. To understand why the gender gap in orgasms continues in the face of overwhelming evidence that women’s orgasms are achievable through sexual practices such as oral sex, we outlined three forms of gender labor from our qualitative data that explain how both women and men understand their sexual practices in ways that perpetuate gender differences in orgasms. First, we showed how both men and women draw on essentialist understandings of gender and heterosexuality to portray men’s orgasms as natural and women’s orgasms as special. Then we examined participants’ heteronormative definitions of what it means to “have regular sex,” in which only men’s orgasms are seen as central and necessary to heterosexual sexual encounters. Last, we uncovered how some women perform gender labor to attribute their lack of orgasms to their own morality and negative feelings about sexual activities that are focused on women’s pleasure.

While our qualitative findings focus on the labor that our participants do to explain the reasons that women in heterosexual couples have fewer orgasms, we argue that this discursive work translates into the gender labor performed *during* sexual practices that perpetuates the gender gap in orgasms. Paula England (2010, 155) argues that persistent gender essentialism has stalled the gender revolution, encouraging women to follow gender-typical pathways in their work and family lives. Norms that promote male dominance persist, especially in the “personal” realm. Our findings point to how gender essentialism, heteronormativity, and moral imperatives are also present in the intimate realm of the bedroom to perpetuate gender inequalities that privilege heterosexual men’s pleasure over women’s. Although both women and men express a desire for women partners to orgasm, the internalized meanings adults have about sex and sexuality influence how they understand and feel about sex. This limits the types of sexual behavior that couples engage in. Our research shows that even in their most intimate interactions, women self-regulate their sexual expression. Similar to Lamont’s (2014) study on the persistence of gender beliefs that reinforce gender differences in dating practices and expectations, we find that gender also shapes men’s and women’s embodied feelings and sexual expression in their sex lives.

There is a sense in our broader popular culture that sexual norms for women have changed dramatically. Our research suggests that we still must move past a taboo about women’s sexuality. Discomfort with their own sexual pleasure and embodied shame lead women to rein in their sexual appetite. Women as a group feel less entitled to the types of sex that lead them to orgasm, relative to men. Even in the most private, intimate settings, our findings show that gender and heteronormativity shape how individuals act.

These findings shine a light on the gender labor that women and men do to make sense of certain sexual practices. Their shared meaning of what counts as regular sex perpetuates the sexual double standard in which orgasms are seen as necessary for men but not for women. Research has demonstrated how this pernicious double standard continues to shape young men’s and women’s hookup behaviors where sexual scripts focus on the need for women, but not men, to guard and/or redeem their reputation (Bogle 2008; Hirsch and Khan 2020; Reid, Elliott, and Weber 2011; Wade 2017). Our research further demonstrates that gendered meanings of orgasm perpetuate a sexual double standard among adults of all ages based on the idea that “regular sex” is focused on vaginal–penile intercourse that requires men to orgasm but not women. Thus, the persistence of the gender gap in orgasms is attributed to not only the fact that men and women are not participating in practices that facilitate women’s orgasms, but that the very understandings of what counts as sex leaves women at a disadvantage that makes their orgasms appear to be “work” and “extra.”

Although much of the literature on gender gaps shows that women have changed much more than men (e.g., England 2010), our research on the continued gender gap in orgasms points to the ways that both heterosexual women and men perform gender labor to explain why women’s orgasms are something extra and not part of regular sex. Much as Jane Ward (2020, 1) has called for the need to challenge the “tragedy of heterosexuality” for straight people—and especially for straight women—who must live under the weight of heteronormativity and male privilege that creates and perpetuates sexual violence and misogyny, our findings demonstrate the need to challenge the shared heteronormative meanings of what counts as sex for both women and men.

## Notes

1. In our analyses of heterosexual encounters, we rely on the gender of one’s most recent sexual partner rather than sexual identity, which does not always convey the gender of each sexual partner. Similarly, our interview participant pool consists of 37 self-identified heterosexual, one bisexual, and two gay individuals. We include responses from all of our participants about their beliefs and perspectives on gender dynamics, sexual practices, and orgasms, because these provided important insights into orgasms within heterosexual sex (see Table 3).
